# The Surgical Treatment of Dysentery*Paper read before South Carolina Medical Society, April, 1895.

**Published:** 1896-03

**Authors:** E. L. Patterson

**Affiliations:** Barnwell, S. C.


### THE SURGICAL TREATMENT OF DYSENTERY.*

By E. L. PATTERSON, M.D.,

BARNWELL, S. C.

Perhaps no disease which we are called upon to treat is more unsatisfactory, so far as the results of the treatment employed, than the one under consideration.

Until recent years little or no advance has been made in the treatment of dysentery; but advancing science, with the introduction of antiseptics, and a better knowledge of hygiene, has done much to eliminate this disease. It is one of the oldest known diseases in its clinical history. Hippocrates, 430 B. C., made frequent reference to the disease, and the ancient writers observed that it was a disease of spring and summer, and in some way was connected with heat and moisture.

Opium, at this period, was used extensively in the treatment of this disease, and was considered the "sheet anchor." It was used in the form of the juice of the poppy. By the time of Galen, it was used to such an extent as to call for protest against its abuse.

Sydenham, in 1669, was an ardent advocate of the opium treatment, but prefaced it with a laxative. He also flushed the rectum with liquids, all of which might be considered good treatment at the present day.

Zimmerman, in 1720, discarded venesection, and was among the first to recognize the value of ipecac, and objected strenuously to the use of opium until the alimentary canal was cleared by appropriate cathartics. This marks an important era in the treatment of this disease.

---

* Paper read before South Carolina Medical Society, April, 1895.

A writer of repute has said that dysentery was pre-eminently a disease of army life, its victims, among soldiers, numbering more than all the other diseases put together. It has followed the tracks of all the great armies which traversed Europe during the Continental wars of the past two hundred years.

It decimated the French, Prussian, and Austrian armies in 1792, and in our late civil war camp dysentery played an important rôle, causing a large death-rate among the soldiers and a still greater among the prisoners.

It is not my intention to go into the clinical history of this disease to any great extent, but simply to call attention to a mode of treatment which, as far as I have been able to learn, has never been employed by the profession or others, and which my limited experience has proven a specific, and to which I have seen fit to apply the term "Surgical."

In my locality, every spring and summer, we have an epidemic of dysentery, beginning, usually, among the negroes, but extending likewise to the whites. In these epidemics I have had ample opportunity for study.

I observed that there was a spasmodic contraction of the anus, and this condition, I thought, was due to the irritation in the rectum, which excites the sphincters into firm contraction. The contracted condition of the sphincters, I concluded, prevented a complete evacuation of the contents of the rectum, and thereby produced the tormina and tenesmus so characteristic of this disease. Having reached these conclusions, I determined to try dilatation of the sphincters and operated as for fissure *in ano*. The result was so gratifying that I have continued this mode of treatment, and now, immediately upon seeing a case, I dilate both the internal and external sphincters, but not quite to the extinct as for fissure *in ano*. It is my custom to give a little chloroform, though I have operated on quite a number without doing so.

The fever which accompanies dysentery is not the result of any inflammatory conditition which exists in the rectum, for in the puerperal condition, we have a more inflamed condition, and yet no fever unless from sepsis; and we do not anticipate fever in our surgical operations unless from lack of antiseptics, therefore we must conclude that the source of fever in dysentery is the result of septic absorption.

The after-treatment consists in irrigating the rectum with antiseptics and astringent solutions, although boiled warm water, one quart, with the addition of two drachms of powdered borax, has given me as good results as anything else. After the dilatation of the anus the dysenteric evacuations cease.

The internal treatment consists of the usual remedies, calomel, salol, and ipecac, in small doses, repeated hourly, until the physiological action of the calomel and ipecac is obtained, followed by a saline cathartic, being my preference, and, if necessary, morphine, gr. $\frac{1}{4}$, atropine, gr. $\frac{1}{150}$, hypodermically, to relieve pain.

The diet should consist of broth and raw eggs. As our water in the country is all surface water, I order boiled water for drinking purposes.

The sanitary surroundings should be looked after and all of the evacuations should be disinfected.

The room should be well ventilated and the bedding frequently changed.

[In the discussion which followed the reading of this paper, Dr. Simon Baruch, of New York, said: I have come from New York for the purpose of adding to my stock of knowledge; I have been here fifteen minutes, and have already learned something profitable. The original method of treating dysentery, just explained by the author, appeals to me as one dictated by common sense; it fulfills all the demands of modern surgical ideas. Common sense is, alas, but too infrequent in medicine, although nowhere may it be applied to greater advantage. The author has omitted, perhaps for the sake of brevity, what I regard as the true principle of his method;—drainage is, next to asepsis, the great promoter of the cure of inflammation, because it removes the products of bacteria and inhibits their multiplication. The lower part of the colon being involved in dysentery, we have a cavity which is practically closed, and, therefore, all these products are incarcerated more or less. Indeed, we may compare this condition to that which exists in an infected stump, whose skin-flaps have healed, leaving a small opening through which the pus is oozing. What does the modern surgeon do? He lays the flap open freely to drain the stump and irrigates it; healing at once begins. Now, when Dr. Patterson paralyzes the sphincter, which closes up the rectum and colon like a purse-string, he allows free exit, without tenesmus, which prevents it, to the mucus, pus, and bacteria which accumulate in dysentery; he then irrigates the bowel without difficulty; he does it as thoroughly as the surgeon does the stump, and this is the reason of his success. It gives me pleasure to commend a therapeutic measure, which is not based upon empirical grounds alone, but whose rationale may be so clearly explained upon principles which have been accepted by the whole medical world. If the method is original (and I have no reason to doubt it), your Association may be congratulated upon its being promulgated by one of its members.]

---

### Strychnine in Pregnancy.

Olenyn has successfully used strychnine in sixteen cases for the correction of weak labor-pains, in doses of 1–32 to 1–25 grain twice daily, at intervals, during the last six or eight weeks of pregnancy. Four of these cases were anemic primiparæ from nineteen to thirty-two years of age, with weak muscles; three were multiparæ under thirty years, with habitual weak labor-pains; four suffered from chronic metritis and had been pregnant at intervals of from three to twelve years; one patient had a small uterine fibroid; two had flabby uterus and relaxed abdominal walls; one had tertiary syphilis and general debility, and another diseased appendages with hysteria. In two primiparæ the forceps had to be used, and in one the child was dead; but in all the other cases delivery was rapid and regular and the children lived. The third stage lasted from ten to twenty minutes and *post-partum* contraction of the uterus was excellent.—*Kansas City Medical Record.*

---

The suprapubic method of lithotomy seems so thoroughly to have established itself among surgeons that one may easily look forward to its being the operation of the future.—Heath, *Medical Record.*

